# Timely Treatment and No Change in Thyroid Cancer Mortality During COVID-19 Pandemic. Reply to Nocini et al. No Impact of COVID-19 Pandemic on Early Mortality for Thyroid Cancer in the US. Comment on “Lee et al. Impact of the COVID-19 Pandemic on Thyroid Cancer Surgery. *Curr. Oncol.* 2024, *31*, 3579–3590”

**DOI:** 10.3390/curroncol31100467

**Published:** 2024-10-17

**Authors:** Max L. Lee, Michelle M. Chen

**Affiliations:** 1 Stanford University School of Medicine, Stanford, CA 94305, USA; maxlee12@stanford.edu; 2 Department of Otolaryngology--Head & Neck Surgery, Stanford University, Palo Alto, CA 94304, USA

We thank Nocini et al. for their thoughtful and timely comment on our paper “Impact of the COVID-19 Pandemic on Thyroid Cancer Surgery” [1,2]. Nocini et al. utilized a large nationwide dataset, the CDC (US Centers for Disease Control and Prevention) WONDER (Wide-Ranging, Online Data for Epidemiologic Research) database and found that between 2018 and 2022, there were no significant differences in age-adjusted mortality rates for thyroid cancer. We believe that the findings of Nocini et al. address an important question that we did not directly address in our paper—whether the early COVID-19 pandemic affected short-term thyroid cancer-specific mortality. Their findings complement the findings from our paper, which found that the early COVID-19 pandemic did not lead to a significant increase in treatment times for thyroid cancer. Overall, we believe that the analysis performed by Nocini et al. suggests that hospitals were able to prioritize high-risk thyroid cancer patients and maintain treatment times and quality of care during the COVID-19 pandemic [1,2].

As hospitals continue to recover from the COVID-19 pandemic and cancer care returns to pre-pandemic levels, it is important that treatment quality and timeliness of care continue to be maintained, while continuing to minimize the overdiagnosis of thyroid cancer. Our paper found that from 2004 to 2019, there was a general trend of both an increased number of diagnoses and a longer time to treatment for thyroid cancer, and then a sharp decrease in both the number of diagnoses and time to treatment from 2019 to 2020 [1]. The findings of Nocini et al. and others suggest that at least in the short-term, a decrease in the number of thyroid cancer diagnoses did not necessarily translate to increased short-term cancer-specific mortality [2]. This may be in part due to the continued timely treatment of advanced stage thyroid cancer, but also suggests that the decreases in the number of new thyroid cancer diagnoses during the pandemic were primarily decreases in the overdiagnosis of clinically insignificant thyroid cancer. Our study demonstrated that the largest decrease in diagnoses was among those with cT1 thyroid cancer [1].

In conclusion, these studies suggest that a decrease in the diagnosis of early-stage thyroid cancer and the timely treatment of advanced thyroid cancer may have allowed early thyroid cancer mortality to remain stable over time. We suggest leveraging the information learned from the COVID-19 pandemic in terms of reducing the work-up of incidental thyroid nodules and treatment prioritization of advanced thyroid cancer as a way to help minimize the overdiagnosis and overtreatment of thyroid cancer in the future.
